# Omics and Male Infertility: Highlighting the Application of Transcriptomic Data

**DOI:** 10.3390/life12020280

**Published:** 2022-02-14

**Authors:** Temidayo S. Omolaoye, Victor A. Omolaoye, Richard K. Kandasamy, Mahmood Yaseen Hachim, Stefan S. Du Plessis

**Affiliations:** 1 Department of Basic Sciences, College of Medicine, Mohammed Bin Rashid University of Medicine and Health Sciences, Dubai 505055, United Arab Emirates; temidayo.omolaoye@mbru.ac.ae (T.S.O.); richard.kandasamy@mbru.ac.ae (R.K.K.); mahmood.almashhadani@mbru.ac.ae (M.Y.H.); 2 Department of Artificial Intelligence and Intelligent Systems, Faculty of Digital Engineering, University of Potsdam, 14469 Potsdam, Germany; adelakunvictoromolaoye1@gmail.com; 3 Centre of Molecular Inflammation Research (CEMIR), Department of Clinical and Molecular Medicine (IKOM), Norwegian University of Science and Technology, 17491 Trondheim, Norway; 4 Division of Medical Physiology, Faculty of Medicine and Health Sciences, Stellenbosch University, Tygerberg 7505, South Africa

**Keywords:** male infertility, omics, genomics, transcriptomics, proteomics, metabolomics

## Abstract

Male infertility is a multifaceted disorder affecting approximately 50% of male partners in infertile couples. Over the years, male infertility has been diagnosed mainly through semen analysis, hormone evaluations, medical records and physical examinations, which of course are fundamental, but yet inefficient, because 30% of male infertility cases remain idiopathic. This dilemmatic status of the unknown needs to be addressed with more sophisticated and result-driven technologies and/or techniques. Genetic alterations have been linked with male infertility, thereby unveiling the practicality of investigating this disorder from the “omics” perspective. Omics aims at analyzing the structure and functions of a whole constituent of a given biological function at different levels, including the molecular gene level (genomics), transcript level (transcriptomics), protein level (proteomics) and metabolites level (metabolomics). In the current study, an overview of the four branches of omics and their roles in male infertility are briefly discussed; the potential usefulness of assessing transcriptomic data to understand this pathology is also elucidated. After assessing the publicly obtainable transcriptomic data for datasets on male infertility, a total of 1385 datasets were retrieved, of which 10 datasets met the inclusion criteria and were used for further analysis. These datasets were classified into groups according to the disease or cause of male infertility. The groups include non-obstructive azoospermia (NOA), obstructive azoospermia (OA), non-obstructive and obstructive azoospermia (NOA and OA), spermatogenic dysfunction, sperm dysfunction, and Y chromosome microdeletion. Findings revealed that 8 genes (*LDHC, PDHA2, TNP1, TNP2, ODF1, ODF2, SPINK2, PCDHB3*) were commonly differentially expressed between all disease groups. Likewise, 56 genes were common between NOA versus NOA and OA (*ADAD1, BANF2, BCL2L14, C12orf50, C20orf173, C22orf23, C6orf99, C9orf131, C9orf24, CABS1, CAPZA3, CCDC187, CCDC54, CDKN3, CEP170, CFAP206, CRISP2, CT83, CXorf65, FAM209A, FAM71F1, FAM81B, GALNTL5, GTSF1, H1FNT, HEMGN, HMGB4, KIF2B, LDHC, LOC441601, LYZL2, ODF1, ODF2, PCDHB3, PDHA2, PGK2, PIH1D2, PLCZ1, PROCA1, RIMBP3, ROPN1L, SHCBP1L, SMCP, SPATA16, SPATA19, SPINK2, TEX33, TKTL2, TMCO2, TMCO5A, TNP1, TNP2, TSPAN16, TSSK1B, TTLL2, UBQLN3*). These genes, particularly the above-mentioned 8 genes, are involved in diverse biological processes such as germ cell development, spermatid development, spermatid differentiation, regulation of proteolysis, spermatogenesis and metabolic processes. Owing to the stage-specific expression of these genes, any mal-expression can ultimately lead to male infertility. Therefore, currently available data on all branches of omics relating to male fertility can be used to identify biomarkers for diagnosing male infertility, which can potentially help in unravelling some idiopathic cases.

## 1. Introduction 

Infertility affects 15% of couples of reproductive age, from which, 50% of the total cases are attributed to the male factor [1], and of these, about 50% are idiopathic. In addition to medical history and physical examination, male infertility is diagnosed mainly through semen analysis and hormonal investigations [2,3]. Due to diversity in semen parameters with different comorbidities, lifestyle, and abstinence period, amongst other risk factors, supplementary assays, such as anti-sperm antibody test, acrosome reaction test, sperm penetration assays, sperm-zona pellucida binding tests, hyaluronan binding assay, and DNA damage test, have been developed over the years [4,5,6]. Semen analysis work remains fundamental, yet inadequate, as the understanding of the underlying etiologies of male infertility remains limited.

The importance of hormonal regulation in the study of male infertility has been highlighted [7,8], especially in the complex process of spermatogenesis. In light of this, some review articles have provided a detailed explanation of how hormone dysfunction impairs male fertility [9,10], thereby re-emphasizing the significance of hormone homeostasis. Male infertility is a multifaceted disorder comprising of irregularities in multiple genes and their interactions with each other [11]. Making the investigation of the role of epigenetic and genetic modifications in the etiologies of male infertility essential. Epigenetics is the study of heritable modifications in gene function that cannot be explained by changes in DNA sequence [11]. Epigenetic changes affect gene expression in histone tail modifications at some specific amino acid residues. Histones are the fundamental proteins required for packaging the nuclear DNA into the nucleosomes. A post-translational modification of these histone proteins serves as the epigenetic mediator in the sperm cell which regulates the gene expression. Epigenetic changes may also affect DNA methylation at the CpG site, and the small non-coding RNAs (ncRNAs) and chromatin remodeling. The small ncRNAs are present in the sperm nucleus and represent another mechanism of epigenetic control. The ncRNAs including the microRNAs (miRNAs) act by base-pairing with the complementary sequences within the mRNA, thus, resulting in the silencing of that gene [12,13]. The collective investigation of hormonal dysfunction, epigenetic modifications and genetic alteration has provided an approach to deeply assess male infertility, starting from the formation of germ cells.

Genetic abnormalities including chromosomal numerical and structural aberrations have long been implicated to play a role in the etiology of male infertility [14]. Several genetic alterations such as chromosomal rearrangement, replacement, gene mutation and Y chromosome microdeletion have been recognized to play a role in male infertility [15]. Although 30% of male infertility cases are due to genetic abnormalities [15,16], recent molecular advances have revealed the significance of “omics”. 

Omics is a term used for the different disciplines of biology that has the ending suffix-omics. It aims at analyzing the structure and functions of the whole constituent of a given biological function at different levels, including the molecular gene level (genomics), transcript level (transcriptomics), protein level (proteomics) and metabolite level (metabolomics) [17]. With the initiation of these molecular techniques, the importance of genomics, transcriptomics, proteomics, and metabolomics in recognizing or identifying the pathways involved in the pathogenesis of male infertility has improved. The current study aims to give an overview of the available evidence on omics and male infertility and to also utilize publicly available transcriptomic data to identify the different pathways and biological processes that may be involved in the pathogenesis of male infertility.

## 2. A Brief Overview of Omics in the Context of Male Infertility

### 2.1. Genomics 

Genomics is the study of the structure, function, evolution, mapping and editing of all genes (genome), as well as the interactions between these genes with each other and with the environment [18]. A genome is an organism’s complete set of DNA. Every cell in the body contains a complete copy of the approximately 3 billion DNA base pairs, or letters, that make up the human genome. Cells in the body have 46 chromosomes. Chromosomes are condensed DNA, and the DNA embodies the genes, while the genes are encoded to function in various physiological processes [18]. The importance of non-coding genes in different aspects of biology has also been highlighted [19].

The genetic basis of male infertility can be a consequence of chromosomal abnormalities, Y chromosome microdeletion or azoospermia factor (AZF) deletion [20], copy number variations, monogenic, polygenic disorders or gene mutation [21]. Chromosomal abnormalities and Y chromosome microdeletions account for 25% of cases of male infertility with azoospermia [22], suggesting their role in spermatogenic dysfunction.

Each chromosome is made up of two arms, namely, short (p) and long (q) arms, with a constriction point, called the centromere, which is present in the middle. The centromere can be located in different positions and this forms the basis for the four different types of chromosomes (telocentric—not seen in humans), acrocentric (chromosome 13–15, 21, 22, Y), sub-metacentric (2, 4–12, 17, 18, X), and metacentric (1, 3, 16, 19, 20) [23]. The Y chromosome contains a male-determining gene, called the sex-determining region Y (*SRY*) gene, which causes the testes to form in the embryo and result in the development of external and internal male genitalia. The Y chromosome has the Yp and Yq arms with the inclusion of the pseudoautosomal region (PAR), which is located at the distal end of both arms [24]. Y chromosomal abnormalities may be numerical (Klinefelter’s, 46XX, 47XYY, 48XXYY, 48XXXY), structural (dicentric Y), rearrangement and/or microdeletion. During spermatogenesis, germ cell meiosis requires PAR pairing, but changes of PAR copy number variations associated with dicentric Y will result in meiotic arrest. Hence, spermatogenic failure is reported in structural chromosomal abnormalities, such as dicentric Y [25]. Chromosomal translocations are up to 4–10 times more frequently observed in infertile males [26]. 

The prevalence of Y chromosome microdeletion ranges from 10% to 15% in azoospermic men and from about 5% to 10% in oligozoospermic men [27]. Some of the known spermatogenesis-related genes on the Y chromosome are located on the AZF region (Yq11.2); hence, the deletion of the long arm leads to genetic abnormality related to male infertility. AZF genes encode 27 proteins [28], and they play major roles in spermatogenesis. AZF has three known regions (AZFa, AZFb, AZFc), with another region located between AZFb and AZFc (AZFd) [29]. These regions have functional genes that are responsible or play a role in the process of spermatogenesis. Represented in Table 1 is the list of AZF regions, their functional units and the repercussions of a deleted functional unit. Briefly, deletion of ubiquitin-specific protease 9 (*USP9Y*) on AZFa was reported to cause spermatogenic disruption [30,31], while the deletion of Dead H Box 3 on Y (*DDX3Y*) is associated with Sertoli cell-only syndrome and/or hypospermatogenesis [31]. Stahl et al. reported that the deletion of AZFb functional genes *CDY2A* and *HSFY 1* and *2* or the under expression thereof is associated with testicular maturation arrest [32]. While other studies have implicated the role of RNA-binding motif on Y (*RBMY*) in spermatogenesis [25]. AZFc functional genes including *DAZ 1-3, BPY2*, amongst others, have been implicated to adversely affect spermatogenesis when altered [33]. Additionally, studies have showed that the mutation of genes, including *CFTR, ADGRG2, PANK2, SLC9A3, TEX11, DMC1, DNAH6, MAGEB4, MCM8 TEX14, TEX15, ZRCC2, ZMYND15*, amongst others, can also result in male infertility [26], as the mutation of genes that regulate recombination and repair of the genome can lead to meiotic arrest. 

Recently, a study investigated the genome of men with severe oligozoospermia, and non-obstructive azoospermia (NOA) to understand the molecular standpoint of these individuals [26]. Of the 285 patients (oligozoospermia = 48; NOA = 237), 30 (10.5%) presented with chromosomal aberrations such as Klinefelter’s syndrome, inversions, translocation and Y chromosome microdeletion, while 69 patients (24.2%) had monogenic variants related to male infertility. The genes with monogenic variations, such as telomere repeat binding bouquet formation protein 1 (*TERB1*), piwi like RNA-mediated gene silencing 2 (*PIWIL2*), MAGE family member E2 (*MAGEE2*), and zinc finger SWIM-type containing 7 (*ZSWIM7*) were reported to play an essential role in germ cell development. Furthermore, Wang et al. identified two variants in the intraflagellar transport protein 140 homolog *(IFT140*) that caused spermatogenic dysfunction in a patient with severe oligoasthenoteratozoospermia without the patient having any physical abnormalities. The spermatozoa of the patient were however morphologically abnormal, having head and tail defects, and there was an absence of *IFT140* in the neck and mid-piece, which was found on control sperm [40]. *IFT140* is a protein required in the structural development of the axonemal microtubules, which means that *IFT140* is vital in the formation of sperm tail, and thus, sperm motility.

Thus, understanding the genetic causes of male infertility is important for better prognosis, treatment, and the assessment of the risk of transmission of genetic abnormalities through natural or assisted reproductive techniques. 

### 2.2. Transcriptomics

During the transcriptional phase, DNA must be read and transcribed or copied into RNA. The gene readouts are called transcripts and the transcriptome is the collection of all the gene readouts present in the cell [18]. There are various types of RNA, but the main type is the messenger RNA (mRNA), which plays a vital role in making proteins. In this process, mRNA is transcribed from genes, then the mRNA transcripts are sent to the ribosomes. The ribosomes in turn read or translate the sequence of amino acids letters in the mRNA and then assemble them into proteins. DNA can also be transcribed into other types of RNA that do not code for proteins. Such transcripts may serve to influence cell structure and also regulate genes. 

Human reproductive processes are driven by the interaction of diverse proteins, even from the stage of germ cell development. It is therefore important to study the transcription of genes at different levels of germ cell development, maturation, and activation. Hence to better understand the underlying pathophysiological mechanism involved in male infertility, studies have investigated the expression of gene transcription in the testes [41], epididymis, sperm [42] and seminal plasma [43]. 

In their pursuit to unravel whether the presence of testes-specific genes in the seminal plasma can serve as biomarkers to predict the occurrence of spermatogenesis in NOA, Hashemi et al. showed the reduced expression of testes-specific genes such as *ZMYND15, TNP1* and *PRM1* [43]. It was further reported that the expression of these genes was significantly decreased in negative sperm retrieval compared to positive sperm retrieval. Thus, it was suggested that the expression of these genes may have the potential for predicting successful sperm retrieval. Another study evaluated the transcriptomic profile of testicular tissues derived from NOA and obstructive azoospermia (OA) men, in order to determine whether gene products from spermatogenic cells could be detected in the Sertoli-cell only testes (SCOT) [44]. Transcripts specific to immature germ cells such as *UTF1, CD9, DDX4, EPCAM, GFRA1, KIT, LIN28, DMRT, GPR125, UCHL1,* and *NANOG* were detected in 65% of SCOT, with 45% of SCOT showing positive immunoreactivity to *DDX4* in the spermatogonia. This suggests that SCOT may contain immature germ cells and *DDX4* may potentially be involved in the proliferation of cells during spermatogenesis. Gatta et al. evaluated specific molecular pathways causing spermatogenic damage, and they reported the downregulation of several genes related to spermatogenesis and are mainly involved in testicular RNA storage [41]. They also showed that four men diagnosed with idiopathic infertility, who have an absence of AZFc deletion in the peripheral blood, showed no testicular expression of DAZ (one of the main functional units of AZFc). This means that some cases of idiopathic male infertility can be ascribed to genetic mutations, because as shown in the study of Gatta et al., although there was no deletion of the entire AZFc region, there was, however, a mutation of the functional gene unit. Jan et al., following the transcriptomic analyses of the successive germ cell subtypes, reported the unique transcriptions of about 4000 genes that are known to encode for meiotic and post-meiotic phases of spermatogenesis were already present in the pre-meiotic phase [45]. Additionally, cell-type-specific expressions of post-translational regulators were found. This suggests that precursor cells already contain the genes necessary for cellular differentiation. Rolland et al., on the other hand, reported the presence of several long non-coding RNAs in the testicular tissues with full spermatogenesis, and over 20 of these genes were uniquely transcribed during spermatogenesis [46]. Zhang et al. reported the association between long non-coding RNA expression and sperm motility [47]. This shows that (i) spermatogenesis is a complex process involving controlled regulation of different transcriptional factors and that (ii) long non-coding RNAs (lncRNA) are crucial for proper spermatogenesis and sperm function. Several other studies have reported the importance of performing transcriptomic analysis in identifying genes that are necessary for normal spermatogenesis [48,49,50].

Now that studies have identified some of the genes required for normal spermatogenesis and sperm function, a transcriptomic assessment can be performed to identify molecular pathways through which these genes interact and how they are involved in male infertility. Later on in the text, genes involved in male infertility will be highlighted using publicly available transcriptomic datasets, and the pathways in which these genes are involved in this pathology will be explored. 

### 2.3. Proteomics 

Proteomics is an important discipline that can be used to achieve rich information on expressed proteins under specific conditions. This technique is also essential because not all encoded genes are translated into proteins, especially under different pathological states. Proteomics is the study of the sum of all proteins (from an organ, tissue, cell or biofluid), their structure, physiological roles and their regulation under specific conditions [51,52]. Proteins are large, complex molecules that are required for the structure, function and regulation of the body’s tissues and organs, and are also known to orchestrate the biological function of a cell [53].

The results of proteomics include protein expression under diverse conditions, which makes it a useful tool in understanding different pathologies. Since semen is a complex mixture of spermatozoa (originating from the testes), with secretions from the epididymis, seminal vesicles and prostate gland, the proteomic evaluation of this specimen in different conditions will shed light on the underlying factors of the said pathology. 

Sharma et al. reported that proteins that protect against oxidative stress (OS) were present in the seminal plasma of both reactive oxygen species (ROS) positive and ROS negative patients. However, these proteins were either downregulated or oxidatively modified in the ROS positive seminal plasma [54]. They furthermore added in another study that thirty-one proteins were differentially expressed between these groups, where six were significantly decreased and twenty-five were increased in the seminal plasma of ROS positive compared to the negative group, and that the deregulated proteins were associated with protection against OS [55]. Knowing that proteomics can serve as a predictive, detective, comparative and selective tool, Yu et al. analyzed the seminal plasma of donkeys with varying freezability potentials to identify proteins that can help in selecting for optimal sperm cryopreservation [56]. Following analysis, 99 proteins known to be involved in oxidoreductase activity (oxidation-reduction processes) were upregulated in the ejaculates with optimal freezability. This shows that a balance between oxidation and reduction must be maintained for proper sperm functioning even after cryopreservation. Furthermore, these proteins can serve as potential biomarkers for cryotolerance. Additionally, Teke et al. analyzed the seminal plasma of infertile and fertile patients who have undergone varicocelectomy, to identify proteins that are differentially expressed in these conditions [57], and proteins that can also be used as biomarkers for semen quality assessment. Eleven proteins were upregulated in the seminal plasma of fertile patients, especially after varicocelectomy. Emphasis was laid on the upregulation of serine protease inhibitor A 5 (*SERPIN A5*), as its concentration increased by 100-fold in the fertile patients. Therefore, they concluded that *SERPIN A5* can be used as a potential seminal biomarker for semen quality assessment in varicocele-related infertility. Likewise, proteomics has been used in identifying proteins that are vital for energy metabolism in metabolic disorders such as diabetes and obesity [58]. 

Several other studies have highlighted the importance of identifying differentially expressed proteins in the sperm and seminal plasma of fertile and infertile men [59,60,61,62,63,64], indicating that proteomics is a useful tool in the study of infertility. Thus, the identification and quantification of proteins in different diseases such as male infertility can help in understanding the role of these proteins and how they potentially contribute to the pathogenesis of the disease. 

### 2.4. Metabolomics

Metabolomics is the study of the chemical reactions that occur in organisms, tissues or cells. Each reaction produces small compounds, called metabolites, which play critical roles in cell homeostasis. 

The production of metabolites are unique to individuals and can give a snapshot of the state of a biological and physiological process in a cell. Metabolites are the substrates, intermediates and end products of metabolism [65]. Metabolomics signifies a key reflection of a gene and protein expression and a genuine representative of a given phenotype. In lieu of this, Ma et al. analyzed the blood plasma of infertile men with various semen parameter abnormalities, such as teratozoospermia, asthenozoospermia, oligozoospermia and azoospermia, for the discovery of potential biomarkers that may be involved in the pathogenesis, hence, characterizing the metabolic features of semen parameter abnormality-related male infertility [66]. It was reported that the main metabolic alterations seen in these patients with diverse semen parameter abnormality included increased levels of energy-related metabolism (tricarboxylic acid cycle, pyruvate metabolism, glyoxylate and dicarboxylate metabolism, glycine, serine, threonine metabolism and saturated fatty acid metabolism), and increased levels of glutathione metabolism, which is related to OS. 

Additionally, Xu et al. reported that the expression of acylcarnitine was positively correlated to sperm concentration and sperm motility and that metabolites such as isopentenyl pyrophosphate, 2-phosphoglyceric acid and γ-glutamyl-Se-methylselenocysteine were negatively correlated to sperm deformity rate [67]. Another study reported the alteration of numerous metabolic pathways such as citric acid cycle, alanine, aspartate and glutamate metabolism after analyzing the metabolic profile of seminal plasma from NOA and fertile men [68]. Several other authors have highlighted other pathways that may be involved in diverse semen parameter abnormality- related male infertility after profiling the seminal plasma metabolites [69,70,71,72,73,74]. Since metabolic profiling can be used to identify altered metabolic pathways, which can then be traced back to protein expression and function, this phenomenon can help in understanding the pathogenesis of male infertility. Hence, metabolomics is an essential tool for modern reproductive medicine.

## 3. Using Publicly Available Transcriptomic Data to Identify Differentially Expressed Genes Involved in Male Infertility

Since transcriptomic assessment can be performed to identify molecular pathways through which genes interact and how they are involved in male infertility, the following section of this study will discuss the utility of transcriptomics in the identification of differentially expressed genes (DEGs) relative to male infertility, using a publicly available transcriptomic dataset. The pathways/biological processes in which these DEGs are involved will be highlighted and briefly reviewed.

## 4. Search Method

### 4.1. Dataset Selection

The Geo Expression Omnibus (GEO), which is a database for gene expression profiling derived from microarray or RNA-Seq experimental data, was employed to identify datasets used in this study. The search term “male infertility” was used, and 1385 datasets were retrieved. The following filters were thereafter applied, “homo sapiens” “expression by microarray”, and a minimum sample size of n = 5, resulting in 21 datasets. After further vetting, 10 datasets met the inclusion criteria for the analysis. The other 11 datasets were excluded because of undefined control groups (n = 6), duplicate (n = 1), unrelated samples (n = 3, the cumulus oocytes of women whose infertility was due to the male factor), and cryptorchidism (n = 1).

The 10 datasets were classified according to the disease or cause of male infertility. The groups include (i) non-obstructive azoospermia (“NOA”; n = 2), (ii) obstructive azoospermia (“OA”; n = 2), (iii) non-obstructive and obstructive azoospermia (“NOA and OA”; n = 2), (iv) spermatogenic dysfunction (n = 2), (v) sperm dysfunction (n = 1), (vi) Y chromosome microdeletion (n = 1) (Table 2).

To put the classification of the disease groups into perspective, their clinical phenotypes will be briefly discussed. Azoospermia is defined as the absence of sperm in the semen after analyzing two successive samples, and it is prevalent in about 10–15% of infertile men [75]. Azoospermia can be categorized into two types: (i) OA, which accounts for 40% of azoospermic cases, and is caused by the blockage or missing connection in the epididymis, vas deferens, or anywhere along the reproductive tract, while—usually— normal spermatogenesis may occur, and (ii) NOA, which accounts for 60% of azoospermia cases. It occurs due to impaired spermatogenesis, genetic deletions or testicular dysfunction [76]. The etiology of azoospermia can be pre-testicular (endocrine disorders), testicular (Sertoli-cell only syndrome (SCOS), testicular torsion, varicocele, orchitis, toxins), and/or post-testicular (ejaculatory disorders). The latter is primarily seen in OA and can be treated by surgically removing or repairing the blockage. Spermatogenic dysfunction occurs when there is disruption of the spermatogenic processes (spermatogenesis), which may be as a result of injury or damage to the testis, or due to genetic mutations. Sperm dysfunction, on the other hand, occurs as a result of damage occurring to the matured or ejaculated spermatozoa. The pathological phenotype of Y chromosome microdeletion has been discussed in details under the ‘genomics’ section. 

### 4.2. Detection of Common Differentially Expressed Genes (DEGs): Methods

The analysis to retrieve the differentially expressed genes (DEGs) was performed using the GEO query and limma R packages through the GEO2R tool for each dataset. The samples were divided into disease and control groups. Thereafter, grouped samples were analyzed using the following parameters: applying log transformation to the data, applying limma precision weights (vooma), and force normalization. After categorizing the genes according to the False Discovery Rate (FDR), the top differentially expressed probes with FDR <0.05 were selected from each dataset.

The retrieved DEGs in each dataset were cleaned (removal of rows with null value) and intersected as shown below using Python programming language.

- The two datasets in the “NOA” group were intersected for common DEGs.
- The two datasets in the “OA” group were intersected for common DEGs.
- The two datasets in the “NOA and OA” group were intersected for common DEGs.
- The two datasets from “spermatogenic dysfunction” were intersected for common DEGs.
- The common DEGs obtained from “NOA” and “NOA and OA” were intersected.
- The common DEGs obtained from “OA” and “NOA and OA” were intersected.
- The common DEGs obtained from “NOA”, “OA” and “NOA and OA” were intersected.
- The common DEGs obtained from “NOA and OA” were intersected with “sperm dysfunction” DEGs.
- The common DEGs obtained from “spermatogenic dysfunction” were intersected with “sperm dysfunction” DEGs.
- The common DEGs obtained from “spermatogenic dysfunction” were intersected with “Y chromosome microdeletion” DEGs.
- The DEGs obtained from “NOA”, “OA”, “NOA and OA”, “spermatogenic dysfunction”, “sperm dysfunction” and “Y chromosome microdeletion” were intersected for common DEGs.

To explore the mechanistic pathways and association/role of these genes in male fertility, gene enrichment ontology was performed for the common identified DEGs using Metascape (https://metascape.org/gp/index.html#/main/step1; accessed on 28 January 2022). 

## 5. Results 

**Figure 1:**
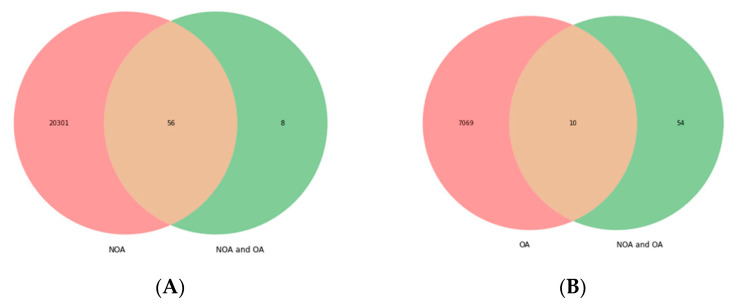

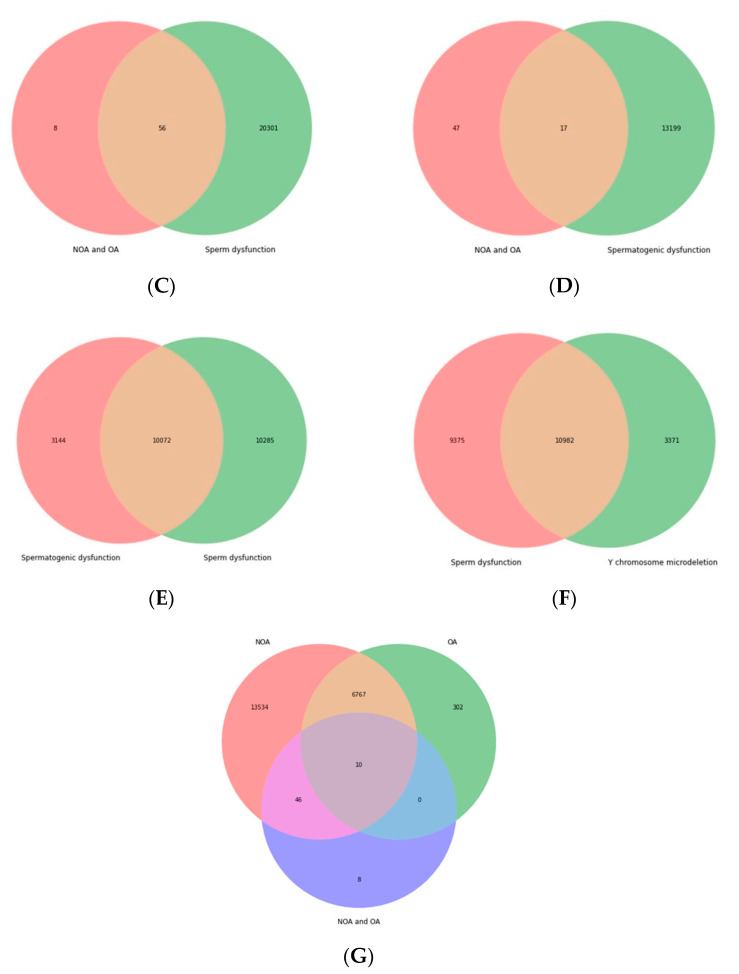
Common differentially expressed genes between the disease groups. (**A**) “NOA” vs. “NOA and OA”; (**B**) “OA vs. “NOA and OA”, (**C**) “NOA and OA” vs. “Sperm dysfunction”; (**D**) “NOA and OA” vs. “Spermatogenic dysfunction”, (**E**) “Spermatogenic dysfunction” vs. “Sperm dysfunction”; (**F**) “Sperm dysfunction” vs. “Y chromosome microdeletion”, (**G**) “NOA” vs. “OA” vs. “NOA and OA”. NOA = non-obstructive azoospermia, OA = obstructive azoospermia, NOA and OA = non-obstructive azoospermia and obstructive azoospermia, SGD = spermatogenic dysfunction, SD = sperm dysfunction, YMD = Y chromosome microdeletion, Count = the number of genes represented in monochrome/frequency, FDR < 0.05.

After analyzing the DEGs across the different disease groups, the following findings were observed. Intersection between the “NOA” and “NOA and OA” groups yielded 56 DEGs (*ADAD1, BANF2, BCL2L14, C12orf50, C20orf173, C22orf23, C6orf99, C9orf131, C9orf24, CABS1, CAPZA3, CCDC187, CCDC54, CDKN3, CEP170, CFAP206, CRISP2, CT83, CXorf65, FAM209A, FAM71F1, FAM81B, GALNTL5, GTSF1, H1FNT, HEMGN, HMGB4, KIF2B, LDHC, LOC441601, LYZL2, ODF1, ODF2, PCDHB3, PDHA2, PGK2, PIH1D2, PLCZ1, PROCA1, RIMBP3, ROPN1L, SHCBP1L, SMCP, SPATA16, SPATA19, SPINK2, TEX33, TKTL2, TMCO2, TMCO5A, TNP1, TNP2, TSPAN16, TSSK1B, TTLL2, UBQLN3*) (Figure 1A). Analysis for common DEGs between “OA” and “NOA and OA” yielded 10 DEGs (*CDKN3, LDHC, ODF1, ODF2, PCDHB3, PDHA2, SPINK2, TNP1, TNP2′*) (Figure 1B); the “NOA and OA” versus “sperm dysfunction” group displayed 56 genes, as shown above (Figure 1C), while intersection between “NOA and OA” and “spermatogenic dysfunction” yielded 17 DEGs (*BCL2L14, CAPZA3, CRISP2, LDHC, ODF1, ODF2, PCDHB3, PDHA2, PLCZ1, ROPN1, ROPN1L, SMCP, SPINK2, TNP1, TNP2, TSSK1B, TTLL2*’) (Figure 1D). The number of common DEGs between “spermatogenic dysfunction” and “sperm dysfunction” is 10,072 (Figure 1E). The intersection between “sperm dysfunction” and “Y chromosome microdeletion” yielded 10,982 genes (Figure 1F), while that of “NOA” versus “OA” versus “NOA and OA” yielded 10 DEGs (Figure 1G). 

**Figure 2:**
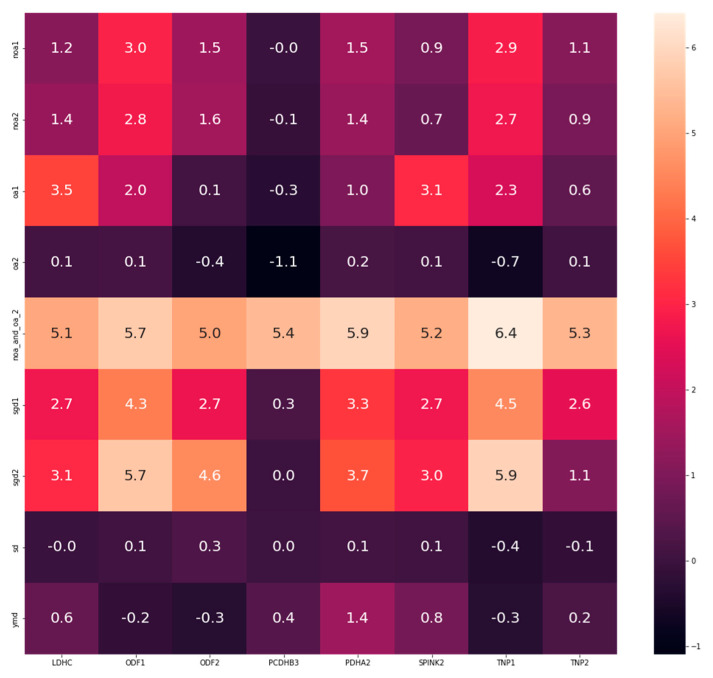
Heatmap of the common differentially expressed genes between all groups using LogFC. Positive value (+) = lower in disease, higher in control; Negative value (−) = higher in the disease, lower in control. NOA = non-obstructive azoospermia, OA = obstructive azoospermia, NOA_OA or NOA_and_OA = non-obstructive azoospermia and obstructive azoospermia, SGD = spermatogenic dysfunction, SD = sperm dysfunction, YMD = Y chromosome microdeletion, LogFC = log fold change, FDR < 0.05.

Interestingly, 8 DEGs (*LDHC, ODF1, ODF2, PCDHB3, PDHA2, SPINK2, TNP1, TNP2*) were found to be common in all groups, i.e., “NOA”, “OA”, “NOA and OA”, “spermatogenic dysfunction”, “sperm dysfunction” and “Y chromosome microdeletion”. A heatmap showing the LogFC of each gene (*LDHC, ODF1, ODF2, PCDHB3, PDHA2, SPINK2, TNP1, TNP2*) against the disease groups is presented in Figure 2.

Following gene enrichment ontology analysis through the Metascape tool, the 8 DEGs common to all groups (“NOA”, “OA”, “NOA and OA”, “spermatogenic dysfunction”, “sperm dysfunction” and “Y chromosome microdeletion”) were shown to be involved in germ cell development, spermatid development, and spermatid differentiation (*ODF2, SPINK2, TNP1, TNP2*), regulation of proteolysis (*SPINK2, TNP1, TNP2*), spermatogenesis (*ODF1, ODF2, SPINK2, TNP1, TNP2*), and metabolic processes (*PDHA2, LDHC*). Additionally, the 56 DEGs obtained between “NOA” versus “NOA and OA” were shown to be involved in different biological processes, such as Spermatogenesis (*ODF1, ODF2, SPINK2, TNP1, TNP2, SHCBP1L, ROPN1L, SPATA16, TSSK1B, C9orf24, RIMBP3, CABS1, GTSF1, ADAD1, CFAP206, GALNTL5, SPATA19*), sperm motility (*LDHC, SMCP, TNP1, ROPN1L, CABS1, CFAP206*), and DNA conformation change (*TNP1, TNP2, BANF2*). The full process list is presented in Table 3. 

**Figure 3:**
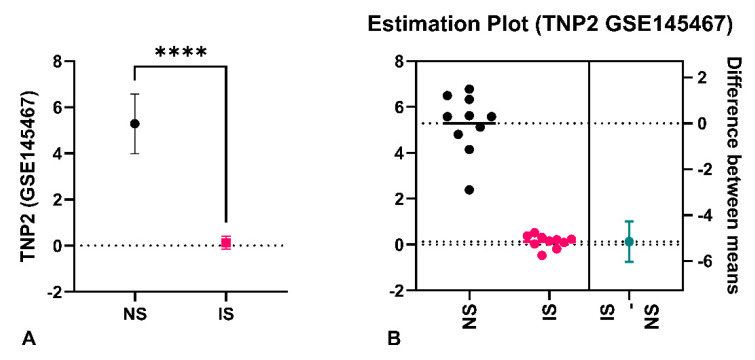
*TNP2* expression in normal and impaired spermatogenesis. (**A**) Difference in *TNP2* expression, (**B**) Estimation plot for *TNP2* expression. **** *p* < 0.00001, NS = normal spermatogenesis, IS = impaired spermatogenesis, IS vs. NS = impaired spermatogenesis versus normal spermatogenesis. Estimation plot = data analysis that uses a combination of confidence intervals and difference in means and sizes.

Additionally, the current study evaluated the results of two genes (from the eight common DEGs), laying emphasis on the log fold change (LogFC). The two genes (transition nuclear proteins (*TNP1* and *TNP2*) were analyzed and their LogFC across all datasets are represented in Table 4. To validate whether the LogFC truly represents the status of the gene expression, the level of testicular *TNP2* was determined from a study that investigated transcriptome changes in patients with severely impaired spermatogenesis (n = 10) versus patients with normal spermatogenesis (n = 10) (GSE145467). After applying filters of interest as stated in the methods section, with significance accepted at <0.05, the level of *TNP2* expression was significantly reduced in the impaired spermatogenesis group compared to the normal group. The mean ± standard deviation is 0.1286 ± 0.2860 vs. 5.285 ± 1.294; *p* < 0.0001 respectively (Figure 3). Validating the result, the LogFC for *TNP2* from dataset GSE145467 (which was used for the analysis above) is 5.405928. This means that the testicular expression of *TNP2* is higher in the control group compared to the group with impaired spermatogenesis (i.e., *TNP2* expression was downregulated in impaired spermatogenesis). Following the same trend of analysis, the expression of *PDHA2, LDHC, SPINK2, ODF1, ODF2,* and *PCDHB3* are represented as LogFC in Figure 2.

## 6. Discussion 

This section will briefly highlight the role of the eight (*TNP1, TNP2, PDHA2, LDHC, SPINK2, ODF1, ODF2,* and *PCDHB3*) common DEGs in male infertility as obtained from our findings.

### 6.1. Transient Nuclear Proteins (*TNPs*)

Transient nuclear proteins (*TNPs*) are proteins that replace nuclear histones and subsequently lead to the substitution by protamine (PRM) during spermiogenesis. During spermiogenesis, the sperm nucleus undergoes evident rearrangement, which involves the removal of histones and their replacement by numerous nuclear proteins, including the *TNPs* [77,78]. *TNPs* aid the remodelling of chromatin structure [77]. Studies have shown that mouse null mutants for either *TNP1* or *TNP2* became subfertile [79,80], while Zhao et al. (2004) reported that mice that lack both *TNPs* became infertile [81]. A study was carried out on *TNP1* and *TNP2* gene sequencing from blood samples of 282 sterile men, 270 men with *TNP1* deficiency and 266 proven fertile men [78]. Five amino-acid substitutions causing nucleotide polymorphisms in the open reading frame of the *TNP2* gene were observed. Deletion of 15 nucleotides which encompassed the recognition site for cAMP response element transcription factor was found in the 5-forward promoter region of the *TNP1* gene in infertile men [78]. This means that the deletion reduces *TNP1* expression and may cause male infertility. Another study that investigated the expression of *TNPs* in the sperm samples of smoking men, reported the down regulation of both *TNP1* and *TNP2* in the spermatozoa of men that smoke [82]. Additionally, Venkatesh et al. investigated whether nucleotide variations in *PRM* and *TNP* genes influence sperm DNA integrity and male fertility. After analyzing *PRM* and *TNP* gene nucleotide variations and sperm DNA integrity of 100 oligozoospermic infertile men, 7 nucleotide variations including two novel changes, a non-synonymous mutation in the exon-2 of *PRM2* gene (c.443C > A) and a novel insertion of T (c.396_397InsT) at the 3’UTR region of *TNP1*, were detected [83]. This is supported by another study that reported the adverse effect of *TNP* and protamine mutation on male fertility [84], suggesting that nucleotide variation in *TNP* may be another cause of male infertility. One of the recent studies on the integration and gene co-expression network analysis of scRNA-seq transcriptomes reveals that *C7orf61* and *TNP* can differentiate two round spermatid sub-cellules, further proving the role of *TNP* in spermiogenesis [85].

To further understand the importance of proper chromatin remodeling, which involves histone hyperacetylation and its replacement by *TNPs* and protamine, Eelaminejad et al. investigated the role of Jumonji domain-containing 1A (*JMJD1A*) during this process. *JMJD1A* is a histone H3K9 demethylase, that participates in the transcriptional control of *TNP* and *PRM* genes by demethylating the repressive epigenetic mark of histone H3 lysine 9 in their promoters. The authors reported a severe decrease in *JMJD1A* in the testicular biopsies of men with spermatid maturation arrest and Sertoli cell only syndrome. It was concluded that the low expression of *JMJD1A*, as well as its low incorporation into chromatin in testes with round spermatid maturation arrest, suggests that an inadequate expression of *JMJD1A* might be indicating and/or contributing to round spermatid maturation arrest [86]. Another study reported that azoospermic men with successful sperm retrieval had increased expression of *JMJD1A*, *TNP* and Protamine. This further shows that reduction in the activation of genes responsible for chromatin remodeling may cause impaired sperm elongation, chromatin compaction and sperm DNA integrity [87]. The moral here is that any condition that can lead to the reduction in the expression of *TNPs* genes can impair male fertility. 

### 6.2. Pyruvate Dehydrogenase Component A Gene (PDHA2)

Energy is produced by the sperm mitochondria in form of ATP through oxidative phosphorylation and glycolysis, using the end-product of glycolysis, pyruvate, as the fuel. Pyruvate is generated from diverse cytosolic sources, such as from the oxidation of lactate via lactate dehydrogenase (LDH), transamination of alanine via alanine aminotransferase (ALT) and the terminal product of glycolysis via pyruvate kinase (PK) [88]. Entry of pyruvate into the mitochondrial matrix is mediated by the mitochondrial pyruvate carrier (MPC). Briefly, cytosolic pyruvate is transferred into the mitochondria through the MPC. In the matrix, pyruvate is converted into acetyl CoA or oxaloacetate by pyruvate dehydrogenase (PDH), a complex that is of great importance in the production of ATP. The acetyl CoA produced either enters the citric acid cycle to replenish intermediates such as oxaloacetate or is converted into phosphoenolpyruvate by phosphoenolpyruvate carboxykinase (PECK) [89]. Therefore, in the absence or reduced availability of pyruvate due to the inhibition of the metabolic enzyme complexes, named the pyruvate dehydrogenase complexes (PDC), ATP production may be reduced, and consequently, motility is in turn reduced. PDC has 3 subunits, namely E1 (pyruvate dehydrogenase), E2 (dihydrolipoamide acetyltransferase), and E3 (dihydrolipoamide dehydrogenase) [90]. E1 and E2 generate acetyl-coenzyme, while E3 performs redox recycling [91]. In addition to these 3 subunits, PDC consist of E3 binding protein (E3BP), regulatory kinases such as pyruvate dehydrogenase kinases and pyruvate dehydrogenase phosphatases [90] 

The E1α subunit of the PDC is encoded by the pyruvate dehydrogenase component A gene (*PDHA*), and it has 2 isoforms (*PDHA1* and *PDHA2*) [92]. The *PDHA1* is a X-linked gene, found in the somatic cells and the testis [92], while *PDHA2* is an autosomal gene but is strictly expressed in the testis. *PDHA1* is mostly expressed in the Sertoli cell, followed by the diploid cell and haploid cell, and it is undetectable in the spermatozoa [88,93]. *PDHA2* on the other hand is mostly expressed in the spermatozoa, followed by the haploid germ cell and diploid cell, and it is undetectable in the Sertoli cell. The trend of stage-specific expression of these genes suggests a hypothesis that there is a switch from *PDHA1* to *PDH2* at the meiotic stage of primary spermatocytes. Iannello et al. in the early 90s reported that the stage-specific expression of *PDHA2* is fundamental for spermatocyte differentiation [94,95], and this is supported by several other studies [92,96]. *PDHA2* in the current study was shown to be downregulated in diverse diseases such as spermatogenic dysfunction and sperm dysfunction, indicating that alteration in the normal expression of *PDHA2* may entirely jeopardize male fertility potential. 

### 6.3. Lactate Dehydrogenase C (LDHC)

The lactate dehydrogenase (LDH) catalyzes the reduction of pyruvate to lactate with the concurrent oxidation of NADH to NAD^+^. LDH consists of A and B subunits that assemble into homo- or heterotetramers and are distributed in the body in combinations reflecting the metabolic requirements of different tissues; subunit C is specifically expressed in spermatocytes, spermatids, and sperm. The three subunits (A, B, C) of LDH are encoded by LDHA, LDHB, and LDHC genes [97]. Odet et al. reported in two different studies that the lack of LDHC in mice disturbed glycolysis and hence disrupted sperm ATP homeostasis [98,99]. This was caused by a defect in the renewal of the NAD^+^ cofactor essential for the activity of glyceraldehyde 3-phosphate dehydrogenase, sperm (GAPDHS). The alteration in the expression of *LDHC* may lead to male fertility impairment by disrupting sperm motility, which is a phenotype of sperm dysfunction. 

### 6.4. Protocadherin Beta 3 (PCDHB3)

Although the role of protocadherin beta 3 (*PCDHB3*) has not been fully explored in male infertility, studies have shown their tumour-suppressive properties, however, as they inhibit colorectal cancer cell proliferation, migration and epithelial to mesenchymal transition [100]. Additionally, their role in the modulation of pain [101], neuronal development [101] and cell adhesion [102] have been elucidated. Hence, their role in spermatogenic dysfunction should be investigated.

### 6.5. Serine Protease Inhibitor Kazal-Type 2 (SPINK2)

Serine protease inhibitor Kazal-type 2 (*SPINK2*) belongs to the family of Kazal-type serine peptidase inhibitors (*SPINK*), which have amino acid sequence homology to bovine pancreatic secretory trypsin inhibitor. About 13 members of the SPINK family have been identified, with *SPINK2* being exclusively expressed in the testes [103]. *SPINK2* is shown to be transcribed intensely in the testis and weakly in the epididymis. Their expression is specific to germ cells, as it is evident at the pachytene spermatocyte stage [104]. Lee et al. reported that *SPINK2* mutant mice exhibited significantly impaired fertility, displaying disrupted testicular architectural integrity and compromised spermatogenesis [104]. Studies have also shown that altered (deficiency/decrease) expression of *SPINK2* is associated with NOA [105], azoospermia [106], and overall, male infertility. As reported by Kherraf et al. the mutation of *SPINK2* initiates protease-induced stress, which in turn instigates Golgi Apparatus fragmentation, contributing to the arrest of spermatid differentiation and their shedding from the seminiferous epithelium. Hence, it can be suggested that *SPINK2* is necessary to counteract the action of acrosomal proteases shortly after their synthesis and before they can be safely stored in the acrosome where they remain dormant until their release during the acrosome reaction [106].

### 6.6. Outer Dense Fibers (ODFs)

During spermiogenesis, the nucleus is condensed, the acrosome and the sperm tail are formed, and the excess cytoplasm is discarded. The sperm tail accessory structures develop after the axoneme has been formed, and the fibrous sheath starts to develop along with the principal piece from the tip to the base. The outer dense fibers (ODFs) develop to surround the axoneme in the principal and midpiece. During the last stage of spermiogenesis, mitochondria are assembled helically around the ODFs in the midpiece of sperm tail [107]. Nine ODFs surround the axoneme in the midpiece, of which two are replaced by longitudinal columns of the fibrous sheath which are connected to each other by transverse ribs. These ODFs are encoded by the *ODFs* genes. The current study identified *ODF1* and *ODF2* to be commonly expressed between all disease groups (“NOA”, “OA”, “NOA and OA”, “spermatogenic dysfunction”, “sperm dysfunction” and “Y chromosome microdeletion”). Yang et al. showed that the targeted deletion of *ODF1* resulted in acephalic sperm in homozygous mice of mixed background [108]. Additionally, spermatozoa of *ODF1* mutant mice showed an enlargement in the distance between the nuclear membrane and the capitulum, indicating a weakening of the sperm head-to-tail coupling [108]. ODF2 was shown to interact with the *Cdk5* and *p35* to enhance sperm tail development. *Cdk5* and *p35* are important components of the sperm tail ODFs, as they contribute to the distinct morphology and function of the sperm tail [109]. Overall, *ODF1* and *ODF2* are essential for sperm head-to-tail coupling and may contribute to the proper functioning of the sperm tail. These genes can therefore be investigated in men with NOA.

## 7. Conclusions

The current study has briefly reviewed four important aspects of omics and has furthermore shown the importance of investigating male infertility from genomics, transcriptomics, proteomics, and metabolomics perspectives. The use of applying publicly available transcriptomic data to identify biological processes or pathways that may affect male infertility is also demonstrated. Using the identified DEGs, the maintained physiology of biological processes such as spermatogenesis, spermiogenesis, spermiation, and energy metabolism is crucial for normal fertility potential. In cases where these processes are dysregulated due to the mal-expression of certain genes, male infertility ensues or at least subfertility. Hence, studies investigating male infertility should not only focus on the evaluation of semen parameters but rather on “how” and “why” the semen parameters became abnormal.

## Figures and Tables

**Table 1:** List of AZF subregions and their functional unit. AZFa = azoospermia factor locus a, AZFb = azoospermia factor locus b, AZFc = azoospermia factor locus c.

Subregions	Functional Units	Effects of the Deletion
**AZFa**	i. Ubiquitin-specific protease p on Y (*USP9Y*) ii. Dead/H Box 3 on Y (DBY or *DDX3Y*) iii. Ubiquitous TPR motif on Y (*UTY*)	i. Spermatogenic disruption [31,34] ii. *DDX3Y* is associated with Sertoli cell only syndrome and/or hypospermatogenesis [35]
**AZFb**	i. Chromodomain Y-Linked 1 and 2 (*CDY2A* and *CDY2B*) ii. Heat shock transcription factor, Y-linked 1 and 2 (*HSFY1* and *HSFY2*) iii. RNA-binding motif on Y (*RBMY*)	i. Deletion of *HSFY* or its under expression is associated with testicular maturation arrest [32,36] ii. *RBMY* is expressed in spermatogonia, its deletion may cause maturation arrest [37]
**AZFc**	i. Deleted in azoospermia (*DAZ*) ii. Chromodomain Y 1 (*CDY1*) iii. Basic protein Y 2 (*BPY2*) iv. Testis transcript Y 2 (*TTY2*)	i. Deletion of *DAZ* affects the entire process of spermatogenesis [38]
**AZFd**	i. No candidate gene discovered yet	i. Deletion of the DYS237 locus of AZFd region may impair spermatogenic process [39]

**Table 2:** Classification of the disease groups. NOA = non-obstructive azoospermia, OA = obstructive azoospermia.

Group Name	Accession No	Title	Number of Datasets
NOA	GSE45885	Potential biomarkers of non-obstructive azoospermia identified in microarray gene expression analysis	2
	GSE45887	The gene expression analysis of paracrine/autocrine factors in patients with spermatogenetic failure compared to normal spermatogenesis	
OA	GSE14310	Testicular gene expression profiles in infertile patients with AZFc deletions of the Y chromosome	2
	GSE21391	Comparison of gene expression between a human epididymal cell line derived from the caput epididymidis of a fertile patient and another one derived from the caput epididymidis of an obstructive azoospermic patient	
NOA and OA (NOA_OA)	GSE10886	Spermatogenomics: correlating testicular gene expression to human male infertility	2
	GSE145467	Transcriptome changes in patients with severely impaired spermatogenesis	
Spermatogenic dysfunction (SGD)	GSE4797	Microarray analysis of human spermatogenic dysfunction	2
	GSE6023	Expression data of testis biopsies obtained from men with spermatogenic impairment	
Sperm dysfunction (SD)	GSE26881	mRNA Content of Human Sperm	1
Y chromosome microdeletion (YMD)	GSE21613	Analysis of testicular transcriptome changes in the presence of Y-chromosomal microdeletions	1

**Table 3:** List of biological processes in which the DEGs play a role.

Processes	List of DEGs
Spermatogenesis	*ODF1, ODF2, SPINK2, TNP1, TNP2, SHCBP1L, ROPN1L, SPATA16, TSSK1B, C9orf24, RIMBP3, CABS1, GTSF1, ADAD1, CFAP206, GALNTL5, SPATA19*
Gamete Generation	*ODF1, ODF2, SPINK2, TNP1, TNP2, SHCBP1L, ROPN1L, SPATA16, TSSK1B, C9orf24, RIMBP3, CABS1, GTSF1, ADAD1, CFAP206, GALNTL5, SPATA19*
Spermatid Development	*ODF2, SPINK2, TNP1, TNP2, ROPN1L, TSSK1B, RIMBP3, ADAD1, CFAP206, GALNTL5*
Spermatid Differentiation	*ODF2, SPINK2, TNP1, TNP2, ROPN1L, TSSK1B, RIMBP3, ADAD1, CFAP206, GALNTL5*
Germ Cell Development	*ODF2, SPINK2, TNP1, TNP2, ROPN1L, TSSK1B, RIMBP3, ADAD1, CFAP206, GALNTL5*
Cellular Process Involved In Reproduction In Multicellular Organism	*ODF2, SPINK2, TNP1, TNP2, ROPN1L, TSSK1B, RIMBP3, ADAD1, CFAP206, GALNTL5*
Flagellated Sperm Motility	*LDHC, SMCP, TNP1, ROPN1L, CABS1, CFAP206, KIF2B*
Sperm Motility	*LDHC, SMCP, TNP1, ROPN1L, CABS1, CFAP206*
Microtubule-Based Movement	*LDHC, SMCP, TNP1, ROPN1L, KIF2B, CABS1, CFAP206*
Sperm Chromatin Condensation	*TNP1, TNP2, SMCP, RIMBP3, PLCZ1, BANF2*
Spermatid Nucleus Differentiation	*TNP1, TNP2*
Fertilization	*SMCP, TNP1, TNP2, RIMBP3, PLCZ1*
Single Fertilization	*SMCP, TNP1, TNP2, PLCZ1*
DNA Packaging	*TNP1, TNP2, BANF2*
DNA Conformation Change	*TNP1, TNP2, BANF2*
Nucleus Organization	*TNP1, TNP2*
Glycolysis/Gluconeogenesis	*LDHC, PDHA2, PGK2, TKTL2*
Pyruvate Metabolic Process	*LDHC, PDHA2, PGK2*
Carbon Metabolism	*PDHA2, PGK2, TKTL2*
Microtubule Cytoskeleton Organization	*ODF2, TTLL2, KIF2B, CFAP206, CCDC187*
Protein-Containing Complex Disassembly	*TNP1, KIF2B, CAPZA3*

**Table 4:** LogFC of *TNP1* and *TNP2* for all datasets. FDR < 0.05, NA = not available.

Datasets	Disease Group	*TNP1*	*TNP2*
**GSE45885**	NOA	2.8728	1.1250
**GSE45887**	NOA	0.9015	2.7248
**GSE14310**	OA	7.1410	NA
**GSE21391**	OA	−0.06819	0.4189
**GSE10886**	NOA and OA	6.4300	2.5800
**GSE145467**	NOA and OA	6.4069	5.4059
**GSE4797**	Spermatogenic dysfunction	4.5333	2.5526
**GSE6023**	Spermatogenic dysfunction	5.9017	1.0787
**GSE26881**	Sperm dysfunction	−0.3701	−0.1269
**GSE21613**	Y chromosome microdeletion	−0.3139	0.2229

## Data Availability

The GEO Omnibus database was used to retrieve datasets analyzed in this study (https://www.ncbi.nlm.nih.gov/geo/, accessed date 10 December 2021). Please refer to the methods section for detailed information.
